# Demographics, diagnostics, treatment, and outcomes of patients presenting with acute groin hernia: 15-year multicentre retrospective cohort study

**DOI:** 10.1093/bjsopen/zrad091

**Published:** 2023-10-24

**Authors:** Leo R Brown, Danielle R Clyde, Lucy Q Li, Rebecca Swan, Ross C McLean, Dimitrios Damaskos

**Affiliations:** Department of Clinical Surgery, University of Edinburgh, Royal Infirmary of Edinburgh, Edinburgh, UK; Department of Clinical Surgery, University of Edinburgh, Royal Infirmary of Edinburgh, Edinburgh, UK; Department of Otolaryngology, Queen Elizabeth University Hospital, Glasgow, UK; Department of General Surgery, Ninewells Hospital, Dundee, UK; Department of General Surgery, Queen Elizabeth Hospital, Gateshead, UK; Department of Clinical Surgery, University of Edinburgh, Royal Infirmary of Edinburgh, Edinburgh, UK

## Abstract

**Background:**

Groin hernias commonly present acutely in high-risk populations and can be challenging to manage. This retrospective, observational study aimed to report on patient demographics and outcomes, following acute admissions with a groin hernia, in relation to contemporary investigative and management practices.

**Methods:**

Adult (≥18 years old) patients who presented acutely with a groin hernia to nine National Health Service trusts in the north of England between 2002 and 2016 were included. Data were collected regarding patient demographics, radiological investigations, and operative intervention. The primary outcome of interest was 30-day inpatient mortality rate.

**Results:**

Overall, 6165 patients with acute groin hernia were included (4698 inguinal and 1467 femoral hernias). There was a male preponderance (72.5 per cent) with median age of 73 years (interquartile range (i.q.r.) 58–82). The burden of patient co-morbidity increased over the study period (*P* < 0.001). Operative repair was performed in 2258 (55.1 per cent) of patients with an inguinal and 1321 (90.1 per cent) of patients with a femoral hernia. Bowel resection was more commonly required for femoral hernias (14.7 per cent) than inguinal hernias (3.5 per cent, *P* < 0.001) and in obstructed (14.6 *versus* 0.2 per cent, *P* < 0.001) or strangulated (58.4 *versus* 4.5 per cent, *P* < 0.001) hernias. The 30-day mortality rate was 3.1 per cent for the overall cohort and 3.9 per cent for those who underwent surgery. Bowel resection was associated with increased duration of hospital stay (*P* < 0.001) and 30-day inpatient mortality rate (*P* < 0.001). Following adjustment for confounding variables, advanced age, co-morbidity, obstruction, and strangulation were all associated with an increased 30-day mortality rate (all *P* < 0.001).

**Conclusion:**

Emergency hernia repair has high mortality rates. Advanced age and co-morbidity increase both duration of hospital stay and 30-day mortality rate.

## Teaser

Acute groin hernia presentations are a common feature of the emergency general surgery workload. This high-volume, retrospective, observational study shows that patients presenting with groin hernias are often elderly and are becoming increasingly co-morbid. Changing patient demographics are influencing investigation and management techniques as demonstrated in this study.

## Introduction

The lifetime risk of undergoing surgery for a groin hernia is 27 per cent in men and 3 per cent in women^1,2^. In the UK, over 70 000 groin hernia operations are performed annually with approximately 4000 (approximately 5 per cent) of these undertaken as an emergency^3,4^. Symptomatic groin hernias frequently present with complications of their hernia, such as incarceration and intestinal obstruction^1,5,6^. In the emergency setting, patients requiring operative repair are typically older, with a higher burden of co-morbidity, and surgery is often performed out of hours^2,7,8^.

The 2018 International Hernia Guidelines outline recommendations for the emergent treatment of groin hernias^1^. Although conclusions were limited by a lack of RCTs, important observational data highlights the presence of a femoral hernia or being female as risk factors for incarceration or strangulation, as reported by large cohort studies^6,9–11^. The evidence regarding the use of diagnostic tools in the emergency setting is similarly limited. Currently, the International Hernia Guidelines recommend that clinical examination alone is sufficient to identify associated complications, such as incarceration or strangulation, in patients presenting acutely with a groin hernia^1^. Further, radiological imaging, such as computed tomography (CT) scanning, would therefore only be required in selected cases. However, contemporary evidence shows that even within the UK there remains considerable variability regarding diagnostic pathways, particularly with the use of CT^12^. It is thus unclear whether the information gleaned from the use of such additional diagnostic tools does alter patients’ outcomes or influence the shared decision-making process between patient and surgeon.

Rates of morbidity and mortality following surgery are higher amongst older and more co-morbid patients, particularly in the emergency setting^8,13,14^. This is similarly evident in the subgroup presenting acutely with a groin hernia^15–17^. The observed 90-day mortality rate in the recent UK-based Management of Acutely Symptomatic Hernia (MASH) study was almost 5 per cent^12^, a rate so high that National Emergency Laparotomy Audit (NELA) guidance would usually recommend consideration of intensive care and direct consultant anaesthetist and surgeon involvement^8^. Therefore, accurate quantification of such elevated mortality risk, and the potential associated risk factors, is essential for surgeons and patients as part of the shared decision-making process. It may also prompt further investigation into questions such as the optimal operative approach and the use of mesh for emergency repair of groin hernias. The evidence regarding these is similarly sparse^1^.

This 15-year multicentre, retrospective cohort study aimed to report on patient demographics, as well as trends in the investigative and operative management of acute groin hernia admissions in northern England. The authors hypothesize that these emergent presentations are likely to be associated with a considerable risk of death, in particular owing to the advanced age and co-morbidities of the affected population.

## Methods

Data were requested for all emergency admissions under a general surgeon between 1 January 2002 and 31 December 2016 at each of the nine acute NHS Foundation Trusts in the north of England. This group of hospitals serves a population of over 2.5 million people, living across approximately 8500 km^2^. Data were derived from Hospital Episode Statistics (HES) recorded by each NHS trust. Caldicott approval was granted, and patient data irreversibly anonymized before transfer to the authors.

### Data definitions

The study population comprised all patients, aged 18 years and older, admitted acutely with a groin hernia. Patients with groin hernias were identified using the International Classification of Diseases and Related Health Problems, 10th Revision (ICD-10) and OPCS-4 codes were used to identify the subgroup that underwent an operative repair during their admission (*Appendix S1, S2*). The data provided included: patient demographics, co-morbidities, date of admission, duration of hospital stay (LoS), time from admission to operation, and 30-day inpatient mortality rate. In cases where a concurrent diagnostic code was recorded that could potentially be relevant to the presentation (for example intra-abdominal adhesions in the context of bowel obstruction), patients were excluded. This was to minimize the risk of inclusion of a patient presenting acutely with an alternative surgical pathology and a concurrent, long-standing hernia. Postal codes were converted to Index of Multiple Deprivation scores using a validated postcode conversion tool^18^, and this was used to derive quintiles indicative of relative deprivation. Co-morbidity, as measured by the Charlson Co-morbidity Index, was calculated from secondary ICD-10 codes with weightings utilized by the hospital standardized death ratio^19–21^. A weekend was defined as a Saturday, Sunday or a bank holiday. Trust size was delineated using NELA defined quartiles into small/medium or large/very large^22^.

### Outcomes

The primary outcome in this study was 30-day inpatient mortality rate. Secondary outcomes included LoS and rate of bowel resection in the operative cohort.

### Statistical analysis

Data was stored in a standardized spreadsheet and statistical analyses were performed using ‘R: A Language for Statistical Computing’ (R Foundation Statistical Software; Vienna, Austria) with *tidyverse* and *finalfit* packages. Categorical data were summarized using frequencies and percentages; Pearson's chi-square test was then used to assess for differences between the groups. Continuous variables were presented using the median and interquartile range (i.q.r.) with differences between groups determined by the Mann–Whitney *U* test. Multivariable logistic regression was used to adjust for confounding variables. Results were deemed statistically significant if *P* < 0.050.

## Results

A total of 6165 patients were admitted acutely with groin hernias over the 15-year study interval. Of these, 4698 presented with inguinal hernias and 1467 patients with femoral hernias (*Table 1*). There was a male predominance (*n* = 4469, 72.5 per cent) amongst the cohort with a median age at presentation of 73 years (i.q.r. 58–82). There were more males in the inguinal hernia group (88.0 per cent *versus* 12.0 per cent, *P* < 0.001) and more females in the femoral hernia group (77.1 per cent *versus* 22.9 per cent, *P* < 0.001). Inguinal hernias presented at a slightly younger age (71 (i.q.r. 56–81) years *versus* 76 (i.q.r. 65–84) years, *P* < 0.001). Patients’ co-morbidity, as described by the Charlson Co-morbidity Index, was comparable between groups. A greater proportion of femoral hernias presented over a weekend (24.0 per cent *versus* 20.7 per cent, *P* = 0.008) when compared with inguinal hernias. Rates of obstruction and strangulation were significantly higher in patients presenting with a femoral hernia (both *P* < 0.001).

**Table 1:** Baseline demographics

	Inguinal hernia (*n* = 4698)	Femoral hernia (*n* = 1467)	*P* value
Age (years), median (i.q.r.)	71 (56–81)	76 (65–84)	<0.001
**Sex**			<0.001
Male	4133 (88.0)	565 (12.0)	
Female	336 (22.9)	1131 (77.1)	
**Year of admission**			0.001
2002 to 2006	1510 (32.1)	500 (34.1)	
2007 to 2011	1520 (32.4)	522 (35.6)	
2012 to 2016	1668 (35.5)	445 (30.3)	
**Charlson score**			0.135
0–1	4076 (86.8)	1249 (85.1)	
2–4	540 (11.5)	196 (13.4)	
≥5	82 (1.7)	22 (1.5)
**Deprivation quintile**			0.387
1 (most)	909 (22.5)	266 (20.8)	
2	969 (24.0)	289 (22.6)	
3	754 (18.7)	253 (19.8)	
4	578 (14.3)	200 (15.6)	
5 (least)	829 (20.5)	272 (21.2)
**Admission route**			0.091
A&E	1965 (42.7)	653 (45.0)	
GP	1958 (42.5)	614 (42.3)	
Consultant clinic	79 (1.7)	15 (1.0)	
Other	602 (13.1)	169 (11.6)
**Trust size**			0.056
Small / medium	2307 (49.1)	763 (52.0)	
Large / very large	2391 (50.9)	704 (48.0)	
**Season**			0.043
Spring	1151 (24.5)	389 (26.5)	
Summer	1230 (26.2)	339 (23.1)	
Autumn	1243 (26.5)	375 (25.6)	
Winter	1074 (22.9)	364 (24.8)
**Weekend admission**			0.008
No	3727 (79.3)	1115 (76.0)	
Yes	971 (20.7)	352 (24.0)	
**Obstruction**			<0.001
No	3608 (76.8)	663 (45.2)	
Yes	1090 (23.2)	804 (54.8)	
**Strangulation**			<0.001
No	4577 (97.4)	1225 (83.5)	
Yes	121 (2.6)	242 (16.5)	
**Recurrent hernia**			<0.001
No	2225 (86.0)	1283 (97.1)	
Yes	363 (14.0)	38 (2.9)	

Presented as number (percentage) unless stated otherwise. A&E, accident and emergency; GP, general practitioner; i.q.r., interquartile range.

### Demographic changes over time

No changes in the age (*P* = 0.727) or sex (*P* = 0.674) were noted over the 15-year study interval (*Table S1*). The proportion of acute groin hernias that were inguinal in nature increased over time (75.1 per cent to 78.9 per cent, *P* = 0.001). In later years (2012–2016), patients were more co-morbid (*P* < 0.001) with 19.2 per cent of patients having a Charlson score ≥2, compared with only 8.4 per cent at the start of the study. Considering only the subgroup of patients who underwent surgery, the burden of co-morbidity also increased over the study time interval (*P* < 0.001). Admission via accident and emergency became the most common route, with General Practitioner presentations conversely falling (*P* < 0.001). Rates of obstruction significantly decreased over time (2002–2006; 32.9 per cent *versus* 2012–2016; 27.3 per cent, *P* < 0.001), however, the proportion of patients presenting with strangulation of their groin hernia remained similar.

### Investigation and management of groin hernias

**Fig. 1:**
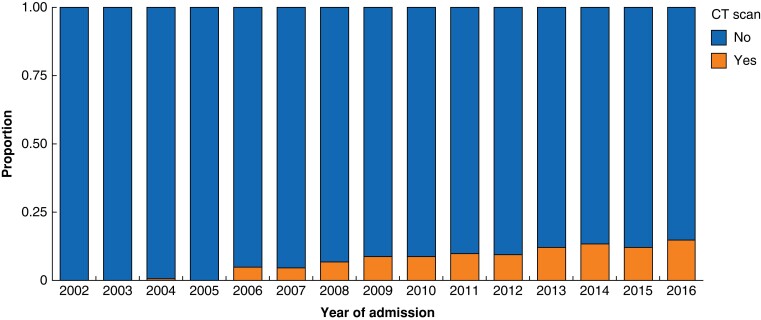
Trends in investigation over time

The use of CT scanning was noted to have increased significantly over the study interval, rising from 1.0 per cent (2002–2006) to 12.3 per cent (2012–2016) (*P* < 0.001, *Fig. 1*). Rates were comparable between femoral and inguinal hernias (*P* = 0.255, *Table S2*). CT scans were more commonly performed in patients who would later be diagnosed with bowel obstruction (8.9 per cent *versus* 6.3 per cent, *P* < 0.001) or strangulation (13.2 per cent *versus* 6.7 per cent, *P* < 0.001). A significantly higher proportion of patients presenting with strangulation or obstruction underwent subsequent operative repair when compared with those patients who did not have a CT scan. Despite CT being used more frequently in patients with complications of their groin hernia, overall, fewer patients underwent operations following CT (55.3 per cent *versus* 64.0 per cent, *P* < 0.001).

A total of 3909 (63.4 per cent) of all patients underwent an emergency repair of their groin hernia. This rate was lower in patients with inguinal hernias (55.1 per cent, *n* = 2588) than those with femoral hernias (90.1 per cent, *n* = 1321) (*P* < 0.001, *Table S2*). Rates of operative management fell during the study interval (2002–2006; 68.6 per cent, *n* = 1378 of 2011 *versus* 2012–2016; 58.3 per cent, *n* = 1231 of 2114, *P* < 0.001). Patients with a Charlson Co-morbidity Index score of ≥2 were more frequently managed non-operatively during their emergent admission, when compared with patients with fewer co-morbidities (15.4 per cent *versus* 12.6 per cent, *P* = 0.010). Most surgeries were performed as open procedures, with only 212 (5.0 per cent) patients undergoing laparoscopic repair. However, an increased use of laparoscopic repair was noted over time (*P* < 0.001, *Fig. S1*), rising from 1.8 per cent (2002–2006) to 6.8 per cent (2012–2016).

Mesh was more frequently used in emergency repair of inguinal hernia (85.5 per cent) when compared with femoral (40.1 per cent, *P* < 0.001). These were utilized with approximately half the frequency when bowel resection was required intraoperatively (femoral: 42.9 per cent *versus* 24.2 per cent, *P* < 0.001 and inguinal: 87.0 per cent *versus* 38.8 per cent, *P* < 0.001).

### Patient outcomes

The overall inpatient 30-day mortality rate was 3.1 per cent and decreased over time (*P* < 0.001, *Table 2*). This rate was significantly higher in those who presented with obstruction (7.9 per cent) or strangulation (15.2 per cent). The mortality rate in patients who were managed operatively increased from 3.9 per cent to 16.1 per cent when a bowel resection was required (*P* < 0.001). The 30-day mortality rate was approximately three-times higher in patients with femoral hernias (6.2 per cent) than inguinal hernias (2.1 per cent). There was no statistically significant difference in the 30-day mortality rate between laparoscopic and open repair (0.6 per cent *versus* 3.9 per cent, *P* = 0.075).

**Table 2:** Outcomes by type of hernia (over time)

	2002–2006 (*n* = 2010)		2007–2011 (*n* = 2042)		2012–2016 (*n* = 2113)	
	Inguinal	Femoral	Inguinal	Femoral	Inguinal	Femoral
Duration of stay (days), median (i.q.r.)	2 (1–5)	5 (3–10)	2 (1–4)	4 (2–8)	2 (1–4)	4 (2–10)
Time to procedure (days), median (i.q.r.)	1 (0–1)	0 (0–1)	1 (0–1)	0 (0–1)	0 (0–1)	0 (0–1)
Postoperative duration of stay (days), median (i.q.r.)	2 (1–5)	4 (2–9)	2 (1–4)	3 (1–8)	2 (1–4)	3 (1–9)
**30-day inpatient mortality rate**						
Alive	1483 (98.2)	465 (93.0)	1474 (97.0)	484 (92.7)	1643 (98.5)	427 (96.0)
Dead	27 (1.8)	35 (7.0)	46 (3.0)	38 (7.3)	25 (1.5)	18 (4.0)

Presented as number (percentage) unless stated otherwise. i.q.r., interquartile range.

Across the study cohort, the median LoS was 2 days (i.q.r. 1–5). This decreased significantly over time in both cohorts (*P* < 0.001). LoS was higher in patients who underwent an operation during their admission (median: 3 (i.q.r. 2–6)) than those who did not (median: 1 (i.q.r. 0–2)). Patients with femoral hernias were operated on earlier but had a longer postoperative inpatient stay (*P* < 0.001, *Table 2*).

Bowel resection was performed in 274 operative cases (11.7 per cent), with the vast majority of these being small bowel resections (95.9 per cent). The rate of bowel resection was greater in femoral hernias (*Table S2*) and in patients who presented with obstruction or strangulation (both *P* < 0.001).

### Adjusted influence on 30-day inpatient mortality rate

**Fig. 2:**
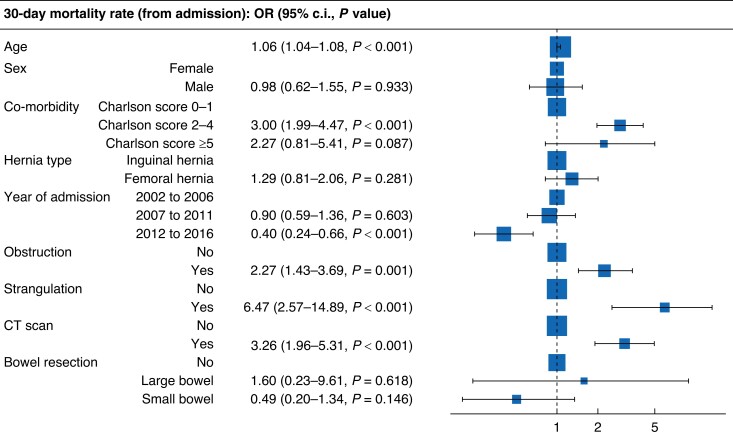
Forest plot for (adjusted) variables associated with 30-day inpatient mortality rate

Following adjustment for confounding variables, both age (OR 1.06 (1.04–1.08), *P* < 0.001) and Charlson score 2–4 (OR 3.00 (1.99–4.47), *P* < 0.001) remained predictors of the 30-day inpatient mortality rate (*Fig. 2* & *Table S3*). While the raw mortality rate was higher for patients presenting with femoral hernias, this did not remain apparent following statistical adjustment (*P* = 0.281). Contemporary admission (2012–2016) was protective (OR 0.40 (0.24–0.66), *P* < 0.001). Both bowel obstruction and strangulation were associated with an adjusted increased mortality rate (*P* < 0.001).

## Discussion

Acute groin hernia presentations are a common feature of the emergency general surgery workload. Despite improvements, the rate of mortality associated with emergency groin hernia presentations remains very high. This study shows that patients presenting with groin hernias are often elderly and are becoming increasingly co-morbid, and both of which are associated with increased LoS and 30-day mortality rate. Imaging has been used more frequently in recent years, and rates of operative repair during index admission are decreasing. CT scanning on admission is being used increasingly for patients presenting with groin hernias. The results of the present study have shown that patients who have a CT scan are three-fold more likely to require a bowel resection. This suggests that preoperative CT is being preferentially used in patients presenting with clinical features indicative of strangulation. Need for bowel resection, particularly colonic resection, is associated with a prolonged hospital admission and increases the 30-day mortality rate from 2.8 per cent to up to 27.3 per cent.

Contemporary international guidance recommends all patients with a symptomatic groin hernia are considered for operative repair^1^. This is based on the evidence that a symptomatic hernia, when not repaired, is more likely to lead to complications, such as strangulation or obstruction, which can ultimately make any future operation more challenging and high risk^23–25^. Older more co-morbid patients are known to be at higher risk of adverse postoperative outcomes^9,10,26^, even in the elective setting. As a result, patients presenting acutely with a groin hernia are often a higher risk group than those undergoing primary elective repair^27,28^. In the present study, 13.6 per cent of patients had a Charlson score greater than or equal to 2 and more than half were aged 70 years and over. Our results also showed that, while no significant change in age at presentation was noted with time, burden of co-morbidity increased significantly over the interval of this study. This finding likely reflects multifactorial population-wide changes in health, including rising obesity rates resulting in increased associated co-morbidities^29,30^.

Emergency surgery of any kind is associated with an increased rate of morbidity and mortality^7,8,10,13^. The overall 30-day mortality rate in our study was 3.1 per cent for all patients presenting acutely with a groin hernia, rising to 3.9 per cent in the subgroup of patients who underwent operative repair. Similar 30-day mortality rates have been reported by other large cohort studies performed by Sweden (2.9 per cent) and Denmark (8 per cent)^10,31^. With very little known about the natural history of an untreated groin hernia, many studies have aimed to identify the clinical characteristics associated with unfavourable outcomes in order to aid clinical decision-making^1,6^. In this study, advanced age and burden of co-morbidity were unsurprisingly both associated with increased LoS and 30-day mortality rate. Interestingly, the 30-day mortality rate of patients requiring bowel resection rose to 16.4 per cent and was as high as 27.3 per cent in those who had resection of their large bowel. This appears to support other previously reported evidence suggesting that delay to surgery, in the presence of acute incarceration and strangulation, is associated with increased bowel resection and adverse patient outcomes^5,6,10,31–33^.

Evidence regarding best surgical practice in this high-risk group is lacking and addressing this knowledge gap represents a step towards improving outcomes. A limitation of this data is its retrospective nature and reliance on accurate clinical coding, which is in turn dependent on accurate documentation by the medical team. The use of a larger data set, such as that of the present study, makes it unlikely that individual errors would influence the results. Furthermore, there is no reason to suspect that any systematic bias should exist in coding data over time. The data were provided to the current group in a pseudo-anonymized form, which prevented the linking of repeated hospital admissions over time. As such, the authors have not been able to comment on the proportion of patients who have had multiple acute admissions or those who have returned on an elective or expedited basis for repair at a later date. There are also limitations regarding the granularity of the data, with details such as the site of incision used for operative repair not being captured through clinical coding. Future studies should look towards prospective or specialist registry data to gain further insight. The findings presented in this study reflect the evolution of contemporary UK-based surgical practices and add to a body of evidence from other population-based European studies^7,10,23,32^.

## Supplementary Material

zrad091_Supplementary_Data

## Data Availability

Caldicott guardian permissions from individual trusts do not permit sharing of data with external sources.

## References

[1] The HerniaSurge Group . International guidelines for groin hernia management. Hernia 2018;22:1–165 10.1007/s10029-017-1668-x PMC5809582 29330835

[2] Primatesta P, Goldacre MJ. Inguinal hernia repair: incidence of elective and emergency surgery, readmission and mortality. Int J Epidemiol 1996;25:835–839 8921464 10.1093/ije/25.4.835

[3] NHS Digital Episode Statistics Data 2016/2017. https://digital.nhs.uk/data-and-information/publications/statistical/hospital-admitted-patient-care-activity/2016-17 (accessed 14 September 2018)

[4] Thousands of groin hernia patients left in pain and at risk of complications by NHS, warn surgeons. Royal College of Surgeons England. July 2018. https://www.rcseng.ac.uk/news-and-events/media-centre/press-releases/hernia-surgery-rationing

[5] Tastaldi L, Krpata DM, Prabhu AS, Petro CC, Ilie R, Haskins IN et al Emergent groin hernia repair: a single center 10-year experience. Surgery 2019;165:398–405 30217396 10.1016/j.surg.2018.08.001

[6] Montgomery J, Dimick JB, Telem DA. Management of groin hernias in adult. JAMA 2018;320:1029–1030 30128503 10.1001/jama.2018.10680

[7] Dahlstrand U, Wollert S, Nordin P, Sandblom G, Gunnarsson U. Emergency femoral hernia repair. Ann Surg 2009;249:672–676 19300219 10.1097/SLA.0b013e31819ed943

[8] NELA Project Team . Seventh Patient Report of the National Emergency Laparotomy Audit December 2019 to November 2020. London, UK: National Emergency Laparotomy Audit, 2021

[9] McGugan E, Burton H, Nixon SJ, Thompson AM. Deaths following hernia surgery: room for improvement. J R Coll Surg Edinb 2000;45:183–186 10881486

[10] Kjaergaard J, Bay-Nielsen M, Kehlet H. Mortality following emergency groin hernia surgery in Denmark. Hernia 2010;14:351–355 20396920 10.1007/s10029-010-0657-0

[11] Koch A, Edwards A, Haapaniemi S, Nordin P, Kald A. Prospective evaluation of 6895 groin hernia repairs in women. Br J Surg 2005;92:1553–1558 16187268 10.1002/bjs.5156

[12] Proctor VK, O’Connor OM, Burns FA, Green S, Sayers AE, Hawkins DJ et al Management of acutely symptomatic hernia (MASH) study. Br J Surg 2022;109:754–762 35608216 10.1093/bjs/znac107

[13] Saunders DI, Murray D, Pichel AC, Varley S, Peden CJ. Variations in mortality after emergency laparotomy: the first report of the UK emergency laparotomy network. Br J Anaesth 2012;109:368–375 22728205 10.1093/bja/aes165

[14] Palmer KL, Law J, Carter B, Hewitt J, Boyle J, Maitra C et al Frailty in older patients undergoing emergency laparotomy: results from the observational ELF study (emergency laparotomy and frailty). Ann Surg 2019;48:ii28–ii29 10.1097/SLA.0000000000003402 31188201

[15] Suppiah A, Gatt M, Barandiaran J, Heng MS, Perry EP. Outcomes of emergency and elective femoral hernia surgery in four district general hospitals: a 4-year study. Hernia 2007;11:509–512 17628736 10.1007/s10029-007-0262-z

[16] Azari Y, Perry Z, Kirshtein B. Strangulated groin hernia in octogenarians. Hernia 2015;19:443–447 24366756 10.1007/s10029-013-1205-5

[17] Ge B-J, Huang Q, Liu L-M, Bian H-P, Fan Y-Z. Risk factors for bowel resection and outcome in patients with incarcerated groin hernias. Hernia 2010;14:259–264 20012331 10.1007/s10029-009-0602-2

[18] English Indices of Deprivation. UK Government. 2015. https://www.gov.uk/government/statistics/english-indices-of-deprivation-2015 (accessed 14 September 2018)

[19] Clinical Indicators Team . Analysis of the Impact of Deprivation on the Summary Hospital-Level Mortality Indicator (SHMI). London: Health and Social Care Information Centre, 2014, 6

[20] Charlson M, Szatrowski TP, Peterson J, Gold J. Validation of a combined comorbidity index. J Clin Epidemiol 1994;47:1245–1251 7722560 10.1016/0895-4356(94)90129-5

[21] Quan H, Li B, Couris CM, Fushimi K, Graham P, Hider P et al Updating and validating the Charlson comorbidity index and score for risk adjustment in hospital discharge abstracts using data from 6 countries. Am J Epidemiol 2011;173:676–682 21330339 10.1093/aje/kwq433

[22] NELA Project Team . NELA Standalone RAG Table. 2018. https://www.nela.org.uk/Fourth-Patient-Audit-Report#pt (accessed 14 September 2018)

[23] Latenstein CSS, Thunnissen FM, Harker M. Variation in practice and outcomes after inguinal hernia repair: a nationwide observational study. BMC Surg 2021;21:45 33472620 10.1186/s12893-020-01030-0 PMC7816298

[24] Hair A, Paterson C, Wright D, Baxter JN, O’Dwyer PJ. What effect does the duration of an inguinal hernia have on patient symptoms? J Am Coll Surg 2001;193:125–129 11491441 10.1016/s1072-7515(01)00983-8

[25] Turaga K, Fitzgibbons RJ Jr, Puri V. Inguinal hernias: should we repair? Surg Clin North Am 2008;88:127–138 18267166 10.1016/j.suc.2007.11.004

[26] Pallati PK, Gupta PK, Bichala S, Gupta H, Fang X, Forse RA. Short-term outcomes of inguinal hernia repair in octogenarians and nonagenarians. Hernia 2013;17:723–727 23307025 10.1007/s10029-012-1040-0

[27] van den Heuval B, Dwars BJ, Klassen DR, Bonjer HJ. Is surgical repair of an asymptomatic groin hernia appropriate? A review. Hernia 2011;15:251–259 21298308 10.1007/s10029-011-0796-y

[28] Wu JJ, Baldwin BC, Goldwater E, Counihan TC. Should we perform elective inguinal hernia repair in the elderly? Hernia 2017;21:51–57 27438793 10.1007/s10029-016-1517-3

[29] Rayman G, Akpan A, Cowie M, Evans R, Patel M, Posporelis S et al Managing patients with comorbidities: future models of care. Future Healthc J 2022;9:101–105 10.7861/fhj.2022-0029 PMC9345245 35928198

[30] Wang YC, McPherson K, Marsh T, Gortmaker SL, Brown M. Health and economic burden of the projected obesity trends in the USA and the UK. Lancet 2011;378:815–825 21872750 10.1016/S0140-6736(11)60814-3

[31] Nilssen H, Nilssen E, Angeras U, Nordin P. Mortality after groin hernia surgery: delay of treatment and cause of death. Hernia 2011;15:301–307 21267615 10.1007/s10029-011-0782-4

[32] Sæter AH, Fonnes S, Rosenberg J, Andresen K. High complication and mortality rates after emergency groin hernia repair: a nationwide register-based cohort study. Hernia 2022;26:1131–1141 35348925 10.1007/s10029-022-02597-8

[33] Sæter AH, Fonnes S, Rosenberg J, Andresen K. Mortality after emergency versus elective groin hernia repair: a systematic review and meta-analysis. Surg Endosc 2022;36:7961–7973 35641700 10.1007/s00464-022-09327-2

