# Erratum to: Demographics, diagnostics, treatment, and outcomes of patients presenting with acute groin hernia: 15-year multicentre retrospective cohort study

**DOI:** 10.1093/bjsopen/zrad157

**Published:** 2023-12-14

This is an erratum to: Leo R Brown, Danielle R Clyde, Lucy Q Li, Rebecca Swan, Ross C McLean, Dimitrios Damaskos, Demographics, diagnostics, treatment, and outcomes of patients presenting with acute groin hernia: 15-year multicentre retrospective cohort study, *BJS Open*, Volume 7, Issue 5, October 2023, zrad091, https://doi.org/10.1093/bjsopen/zrad091

The originally published version of this manuscript has been corrected.

In Table 1, percentages were missing for proportions of male/female for each hernia type. This has been updated.

In addition, in the subsection entitled “Adjusted influence on 30-day inpatient mortality Rate”, the following text required correction:

“Following adjustment for confounding variables, both age (OR 1.06 (1.04–1.08), P < 0.001) and Charlson score 2–4 (OR 3.15 (2.10–4.69), P < 0.001) remained predictors of the 30-day inpatient mortality rate (Fig. 2 & Table S3). While the raw mortality rate was higher for patients presenting with femoral hernias, this did not remain apparent following statistical adjustment (P = 0.202).”

The corrected text now reads as follows:

“Following adjustment for confounding variables, both age (OR 1.06 (1.04–1.08), P < 0.001) and Charlson score 2–4 (OR 3.00 (1.99–4.47), P < 0.001) remained predictors of the 30-day inpatient mortality rate (Fig. 2 & Table S3). While the raw mortality rate was higher for patients presenting with femoral hernias, this did not remain apparent following statistical adjustment (P = 0.281).”

