# Supplementary material for: Demographics, diagnostics, treatment, and outcomes of patients presenting with acute groin hernia: 15-year multicentre retrospective cohort study
Source: BJS Open. 2023 Oct 24;7(5):zrad091. doi: 10.1093/bjsopen/zrad091 (PMC10597656; doi:10.1093/bjsopen/zrad091)
Supplement: zrad091_Supplementary_Data [file zrad091_supplementary_data.docx]

**Trends in Demographics, Management and Outcomes of Patients Presenting Acutely with a Groin Hernia: A 15-Year Multicentre Retrospective Cohort Study**

DR Clyde^1*^, LR Brown^1*^, LQ Li^2^, R Swan^3^, RC McLean^4^ and D Damaskos^1^

1. *Department of Clinical Surgery, University of Edinburgh, Royal Infirmary of Edinburgh, Edinburgh, Scotland.*
2. *Department of Otolaryngology, Queen Elizabeth University Hospital, Glasgow, Scotland*
3. *Department of General Surgery, Ninewells Hospital, Dundee, Scotland.*
4. *Department of General Surgery, Queen Elizabeth Hospital, Gateshead, England.*

**Corresponding author.** Mr. Leo R. Brown, Department of Clinical Surgery, University of Edinburgh, Royal Infirmary of Edinburgh, Edinburgh, Scotland. **Email:** [leorbrown@doctors.org.uk](mailto:leorbrown@doctors.org.uk). **ORCID ID:** <https://orcid.org/000-0001-6181-7020>. **Twitter** @leorbrown

**Supplementary Materials - Index**

| **Supplementary Methods** |  |
| --- | --- |
| Detail **N/A** | *pag. X* |
| Detail **N/A** | *pag. Y* |
| **Supplementary Results** |  |
| Detail **N/A** | *pag. X* |
| Detail **N/A** | *pag. Y* |
| **Supplementary Appendixes** |  |
| Detail **Appendix 1** | *pag. 4* |
| Detail **Appendix 2** | *pag. 5* |
| **Supplementary Figures and Tables** |  |
| Detail **Supplementary Table 1** | *pag. 6* |
| Detail **Supplementary Table 2**  Detail **Supplementary Table 3**  Detail **Supplementary Figure 1** | *pag. 7*  *pag. 8*  *pag. 9* |
| **References** | *pag. Z* |
|  |  |

**Supplementary Appendixes**

**Appendix 1: International Classification of Disease 10^th^ edition (ICD-10) codes used to identify patients with a groin hernia**

| **Inguinal Hernia** | |
| --- | --- |
| **K400** | Bilat inguinal hernia with obstruction without gangrene |
| **K401** | Bilateral inguinal hernia, with gangrene |
| **K402** | Bilateral inguinal hernia, without obstruction or gangrene |
| **K403** | Unilateral or unspecified inguinal hernia with obstruct without gangrene |
| **K404** | Unilateral or unspecified inguinal hernia, with gangrene |
| **K409** | Unilateral or unspecified inguinal hernia without obstruct or gangrene |
| **Femoral Hernia** | |
| **K410** | Bilateral femoral hernia, with obstruction, without gangrene |
| **K411** | Bilateral femoral hernia, with gangrene |
| **K412** | Bilateral femoral hernia, without obstruction or gangrene |
| **K413** | Unilateral or unspecified femoral hernia with obstruct without gangrene |
| **K414** | Unilateral or unspecified femoral hernia, with gangrene |
| **K419** | Unilateral or unspecified femoral hernia without obstruction or gangrene |

**Appendix 2: OPSC Classification of Interventions and Procedures (OPCS-4) Codes Used to Identify Patients with a Groin Hernia**

| **Inguinal Hernia** | |
| --- | --- |
| **T20.1** | Primary repair of inguinal hernia using insert of natural material |
| **T20.2** | Primary repair of inguinal hernia using insert of prosthetic material |
| **T20.3** | Primary repair of inguinal hernia using sutures |
| **T20.4** | Primary repair of inguinal hernia and reduction of sliding hernia |
| **T20.8** | Other specified primary repair of inguinal hernia |
| **T20.9** | Unspecified primary repair of inguinal hernia |
| **T21.1** | Repair of recurrent inguinal hernia using insert of natural material |
| **T21.2** | Repair of recurrent inguinal hernia using insert of prosthetic material |
| **T21.3** | Repair of recurrent inguinal hernia using sutures |
| **T21.4** | Removal of prosthetic material from previous repair of inguinal hernia |
| **T21.8** | Other specified repair of recurrent inguinal hernia |
| **T21.9** | Unspecified repair of recurrent inguinal hernia |
| **Femoral Hernia** | |
| **T22.1** | Primary repair of femoral hernia using insert of natural material |
| **T22.2** | Primary repair of femoral hernia using insert of prosthetic material |
| **T22.3** | Primary repair of femoral hernia using sutures |
| **T22.8** | Other specified primary repair of femoral hernia |
| **T22.9** | Unspecified primary repair of femoral hernia |
| **T23.1** | Repair of recurrent femoral hernia using insert of natural material |
| **T23.2** | Repair of recurrent femoral hernia using insert of prosthetic material |
| **T23.3** | Repair of recurrent femoral hernia using sutures |
| **T23.8** | Other specified repair of recurrent femoral hernia |
| **T23.9** | Unspecified repair of recurrent femoral hernia |

**Supplementary Figures and Tables**

**Supplementary Table 1 - Trends in Demographics Over Time**

|  |  | **2002 to 2006** | **2007 to 2011** | **2012 to 2016** | **p value** |
| --- | --- | --- | --- | --- | --- |
| **Age** | Median (IQR) | 73 (58 - 81) | 73 (59 - 82) | 73 (58 - 81) | 0.727 |
| **Gender** | Female | 540 (26.9) | 574 (28.1) | 582 (27.5) | 0.674 |
|  | Male | 1470 (73.1) | 1468 (71.9) | 1531 (72.5) |  |
| **Hernia Type** | Inguinal Hernia | 1510 (75.1) | 1520 (74.4) | 1668 (78.9) | 0.001 |
|  | Femoral Hernia | 500 (24.9) | 522 (25.6) | 445 (21.1) |  |
| **Charlson Score** | 0-1 | 1841 (91.6) | 1777 (87.0) | 1707 (80.8) | <0.001 |
|  | 2-4 | 150 (7.5) | 235 (11.5) | 351 (16.6) |  |
|  | ≥5 | 19 (0.9) | 30 (1.5) | 55 (2.6) |  |
| **Deprivation Quintile** | 1 (most) | 375 (22.0) | 408 (23.0) | 392 (21.3) | 0.157 |
|  | 2 | 372 (21.8) | 433 (24.4) | 453 (24.6) |  |
|  | 3 | 311 (18.3) | 326 (18.3) | 370 (20.1) |  |
|  | 4 | 261 (15.3) | 252 (14.2) | 265 (14.4) |  |
|  | 5 (least) | 384 (22.5) | 358 (20.1) | 359 (19.5) |  |
| **Admission Route** | A&E | 639 (31.8) | 919 (45.0) | 1060 (52.9) | <0.001 |
|  | GP | 1073 (53.4) | 871 (42.7) | 628 (31.4) |  |
|  | Consultant Clinic | 20 (1.0) | 24 (1.2) | 50 (2.5) |  |
|  | Other | 278 (13.8) | 228 (11.2) | 265 (13.2) |  |
| **Trust Size** | Small / Medium | 970 (48.3) | 988 (48.4) | 1112 (52.6) | 0.006 |
|  | Large / Very Large | 1040 (51.7) | 1054 (51.6) | 1001 (47.4) |  |
| **Season** | Spring | 506 (25.2) | 516 (25.3) | 518 (24.5) | 0.199 |
|  | Summer | 484 (24.1) | 548 (26.8) | 537 (25.4) |  |
|  | Autumn | 564 (28.1) | 507 (24.8) | 547 (25.9) |  |
|  | Winter | 456 (22.7) | 471 (23.1) | 511 (24.2) |  |
| **Weekend Admission** | No | 1569 (78.1) | 1607 (78.7) | 1666 (78.8) | 0.810 |
|  | Yes | 441 (21.9) | 435 (21.3) | 447 (21.2) |  |
| **Obstruction** | No | 1348 (67.1) | 1386 (67.9) | 1537 (72.7) | <0.001 |
|  | Yes | 662 (32.9) | 656 (32.1) | 576 (27.3) |  |
| **Strangulation** | No | 1895 (94.3) | 1916 (93.8) | 1991 (94.2) | 0.801 |
|  | Yes | 115 (5.7) | 126 (6.2) | 122 (5.8) |  |

**Supplementary Table 2 – Investigation and Management of Groin Hernia**

|  |  | **Inguinal Hernia** | **Femoral Hernia** | **p value** |
| --- | --- | --- | --- | --- |
| **CT Scan** | No | 4376 (93.1) | 1353 (92.2) | 0.255 |
|  | Yes | 322 (6.9) | 114 (7.8) |  |
| **Operative Management** | No | 2110 (44.9) | 146 (10.0) | <0.001 |
|  | Yes | 2588 (55.1) | 1321 (90.1) |  |
| **Mesh Repair** | No | 375 (14.5) | 791 (59.9) | <0.001 |
|  | Yes | 2213 (85.5) | 530 (40.1) |  |
| **Recurrent Repair** | No | 2225 (85.9) | 1283 (97.1) | <0.001 |
|  | Yes | 364 (14.1) | 38 (2.9) |  |
| **Operative Approach** | Open | 2751 (95.0) | 1279 (95.0) | 0.459 |
|  | Laparoscopic | 131 (4.5) | 58 (4.3) |  |
|  | Laparoscopic Converted to Open | 13 (0.4) | 10 (0.7) |  |
| **Bowel Resection** | No | 2508 (96.9) | 1127 (85.3) | <0.001 |
|  | Large Bowel | 11 (0.4) |  |  |
|  | Small Bowel | 69 (2.7) | 194 (14.7) |  |

**Supplementary Table 3: Predictors of Thirty Day Inpatient Mortality**

|  |  | **Alive** | **Dead** | **OR  (univariable)** | **OR  (multivariable)** |  |
| --- | --- | --- | --- | --- | --- | --- |
| **Age** | Median (IQR) | 72 (58,81) | 83.0 (77,87) | 1.08 (1.06-1.10, *p*<0.001) | 1.06 (1.04-1.08, *p*<0.001) |  |
| **Gender** | Female | 1605 (94.6) | 91  (5.4) | - | - |  |
|  | Male | 4371 (97.8) | 98  (2.2) | 0.40 (0.30-0.53, *p*<0.001) | 0.98 (0.62-1.55, *p*=0.933) |  |
| **Charlson Score** | 0-1 | 5209 (97.8) | 116  (2.2) | - | - |  |
|  | 2-4 | 671 (91.2) | 65  (8.8) | 4.35 (3.16-5.93, *p*<0.001) | 3.00 (1.99-4.47, *p*<0.001) |  |
|  | ≥5 | 96 (92.3) | 8  (7.7) | 3.74 (1.64-7.42, *p*=0.001) | 2.27 (0.81-5.41, *p*=0.087) |  |
| **Hernia Type** | Inguinal Hernia | 4600 (97.9) | 98  (2.1) | - | - |  |
|  | Femoral Hernia | 1376 (93.8) | 91  (6.2) | 3.10 (2.32-4.16, *p*<0.001) | 1.29 (0.81-2.06, *p*=0.281) |  |
| **Year of Admission** | 2002 to 2006 | 1948 (96.9) | 62  (3.1) | - | - |  |
|  | 2007 to 2011 | 1958 (95.9) | 84  (4.1) | 1.35 (0.97-1.89, *p*=0.080) | 0.90 (0.59-1.36, *p*=0.603) |  |
|  | 2012 to 2016 | 2070 (98.0) | 43  (2.0) | 0.65 (0.44-0.96, *p*=0.034) | 0.40 (0.24-0.66, *p*<0.001) |  |
| **Obstruction** | No | 3387 (98.9) | 37  (1.1) | - | - |  |
|  | Yes | 2589 (94.5) | 152  (5.5) | 9.03 (6.41-13.02, *p*<0.001) | 2.27 (1.43-3.69, *p*=0.001) |  |
| **Strangulation** | No | 5806 (97.3) | 161 (2.7) | - | - |  |
|  | Yes | 170 (85.9) | 28 (14.1) | 7.55 (5.37-10.50, *p*<0.001) | 6.47 (2.57-14.89  *p*<0.001) |  |
| **CT Scan** | No | 5581 (97.4) | 148 (2.6) | - | - |  |
|  | Yes | 395 (90.6) | 41 (9.4) | 3.91 (2.70-5.56, *p*<0.001) | 3.26 (1.96-5.31, *p*<0.001) |  |
| **Bowel Resection** | No | 3536 (97.3) | 99 (2.7) | - | - |  |
|  | Large Bowel | 8 (72.7) | 3 (27.3) | 13.39 (2.90-47.09, *p*<0.001) | 1.60 (0.23-9.61, *p*=0.618) |  |
|  | Small Bowel | 222 (84.4) | 41 (15.6) | 6.60 (4.43-9.66, *p*<0.001) | 0.49 (0.20-1.34, *p*=0.146) |  |

**Supplementary Figure 1: Trends in Operative Approach over Time**

**
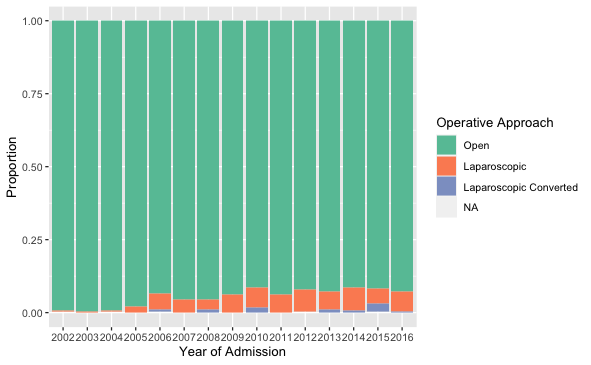
**
